# High Internal Phase Emulsions Stabilized by Pea Protein Isolate Modified by Ultrasound Combined with pH-Shifting: Micromorphology, Rheology, and Physical Stability

**DOI:** 10.3390/foods12071433

**Published:** 2023-03-28

**Authors:** Jingnan Zhang, Siqi Zhao, Linte Li, Baohua Kong, Haotian Liu

**Affiliations:** College of Food Science, Northeast Agricultural University, Harbin 150030, China

**Keywords:** pea protein isolate, high internal phase emulsions, micromorphology, rheological behavior, 3D printing

## Abstract

In this study, the interfacial behavior of high internal phase emulsions (HIPEs), stabilized by ultrasound combined with pH-shifting modified pea protein isolate (MPPI), was investigated, and its emulsification process and stabilization mechanism were discussed. The effects of MPPI concentration on the micromorphology, droplet size, rheology, and stability of HIPEs were investigated. As the MPPI concentration increased, the appearance of HIPEs gradually changed from a relatively fluid state to a plastic solid-like state with detailed texture. There occurred a gradual decrease in droplet size, the cohering of an orderly and tight arrangement, in addition to the formation of a bilayer elastic interface layer. The macro- and microrheological assessments confirmed that the apparent viscosity, storage modulus, elasticity index, and macroscopic viscosity index increased gradually. Furthermore, it was demonstrated that 5 wt% MPPI-stabilized HIPEs had the potential to be used as 3D printing inks. Stability evaluation showed that the TURBISCAN stability index decreased and centrifugal stability increased. The appearance and microstructure remained highly stable after heating at 80 °C for 30 min and storage at 4 ℃ for 90 days. These findings confirm that MPPI improves the rheological behavior and stability of HIPEs by modulating the interfacial adsorption and network structure.

## 1. Introduction

High internal phase emulsions (HIPEs) are highly concentrated emulsion systems with an internal phase volume fraction (φ) higher than 74% [1]. With ideal solid-like appearance, gel-like rheological behavior, and high stability, HIPEs have attracted extensive attention in the fields of food, materials, tissue engineering, medicine, and cosmetics. In the past, HIPEs have been stabilized with large amounts of surfactants (5–50%) or inorganics, which have limited applicability in the food industry because of factors such as cost, health, environmental protection, “clean labelling,” and legal constraints [2]. Gradually, the use of natural biopolymers to stabilize HIPEs has attracted research interest.

Pea protein isolate (PPI), with its high nutritional value and hypoallergenicity, has aroused considerable interest as a sustainable alternative to animal-based protein [3]. However, the highly ordered spherical structure and poor solubility of native PPI inhibit its ability to emulsify at the oil–water interface [4]. Although some studies have demonstrated that treatment of PPI by glycosylation [5], potassium metabisulfite induction [6] and microgel [7] formation enabled it to stabilize HIPEs, these strategies are complex and require precise control of the reaction process. Many studies have used pH shifting, a simple, mild, and effective chemical modification method, to modify protein in order to improve its solubility and emulsification [8]. Under extreme pH conditions, the charged functional groups of amino acids create molecular repulsion, which triggers the irreversible unfolding of the protein structure. If the pH is relocated to a neutral value, the protein structure will transform into a molten spherical state, and the structure at this time will become more flexible, which is conducive to its extension at the oil–water interface [9]. For PPI, however, although pH-shifting drives the formation of molten globules, its naturally rigid structure still hinders its adsorption and positioning at the oil–water interface and it cannot provide good stability and support for the system, which is undesirable for emulsified systems, especially HIPEs.

High-intensity ultrasound (HIU) is a successful physical strategy for disrupting interactions, dispersing aggregates, and modifying the structure of proteins, especially plant proteins [10], to achieve superior functional properties for a variety of formulated foods. The key to material modification by HIU lies in the acoustic cavitation produced during the ultrasonic process [11]. Bubbles generated by acoustic cavitation collapse upon reaching a critical size, thereby increasing the local pressure in the cavitation zone and creating high shear forces and turbulence around the bulk liquid, enabling physical modification of the material [12]. Our group earlier found that HIU treatment can make the conformation of the molten spherical state PPI undergoing pH-shifting more flexible, endowing better solubility and emulsification [4]. A fascinating hypothesis is that the molten state induced by pH-shifting promotes the adsorption of proteins at the interface, whereas the flexible conformation caused by HIU treatment increases the possibility of intertwined interfacial proteins, providing better stability and support for HIPEs.

Hence, the purpose of this study was to provide a strategy to prepare HIPEs using ultrasound combined with pH-shifting modified pea protein isolate (MPPI) based on interfacial emulsification and cross-linked network support. First, various concentrations of MPPI-stabilized HIPEs were characterized, and the dynamic interfacial adsorption and regulation and control distribution process were elucidated. Then, the relationship between the emulsification mechanism and property improvement of MPPI-stabilized HIPE in terms of appearance, micromorphology, rheological behavior, and stability was discussed. Thus, this study developed a new approach to preparing PPI-based HIPEs with desirable viscoelasticity, high plasticity, and high stability and, explored their possible use as a solid fat mimic, providing insights for the development and utilization of PPIs.

## 2. Materials and Methods

### 2.1. Materials

The pea protein isolate (PPI, protein ∼87.31%) used in this study was provided by Northeast Agricultural University (Harbin, Heilongjiang, China). Sunflower oil was purchased from Yihai Kerry Co., Ltd. (Shanghai, China). All the chemicals and reagents used in this study were of analytical grade.

### 2.2. PPI Modified by High-Intensity Ultrasound and pH-Shifting (MPPI)

Similar to our previous work [4], the hydrated overnight PPI (5 wt%) was adjusted to pH 12 using 2 M NaOH, and then treated at 20 kHz with 500 W ultrasonic power for 10 min. The solution was stirred at room temperature for 1 h, after which the pH was adjusted to 7 with 1 M HCl and then the solution diluted to various concentrations (1–5 wt%). If necessary, the pH was fine-tuned to 7 using 0.1 M HCl.

### 2.3. Preparation of HIPEs

PPI or MPPI (1–5 wt%) aqueous solution and sunflower seed oil were mixed in a 3:1 ratio (*v/v*), and 0.02 wt% sodium azide was added to the aqueous phase to prevent microbial growth before mixing. HIPEs (75% internal phase volume fraction) were then formed by shearing through a homogenizer (IKA Ultra-Turrax T18, Staufen, Germany) which operated at 15,000 rpm for 2 min, after which they were stored at 4 °C [13].

### 2.4. Micromorphology

#### 2.4.1. Super-Resolution Microscopy (SRM)

The micromorphology of HIPEs was observed through super-resolution microscopy (OMX SR, Boston, MA, USA). HIPEs of 0.5 g were stained using 20 μL of Nile blue (0.1 wt% in water) and 25 μL of Nile red (0.1 wt% in ethanol) for 30 min in the dark for the water and oil phases, respectively [14]. The excited wavelengths of Nile blue and Nile red were 633 nm and 488 nm, respectively.

#### 2.4.2. Cryogenic Scanning Electron Microscopy (Cryo-SEM)

The micromorphology of HIPEs was assessed via cryo-SEM (Hitachi, Tokyo, Japan) according to the method of Yan et al. [15]. Briefly, samples were frozen-cut in liquid nitrogen, and sublimated HIPEs were sputtered with platinum, and these were then inserted into a cold module chamber for observation.

#### 2.4.3. Optical Microscope

The microscopic morphology of HIPEs was observed using an optical microscope (BX53, Olympus, Japan). A small amount of each sample was smeared onto the glass slide, covered with a coverslip, and observed under a 100× oil lens.

### 2.5. Droplet Size Analysis

The droplet size distribution and mean droplet diameter of the HIPEs were determined using a static light scattering technique (Malvern 3000, Malvern, UK). The samples were diluted with deionized water until they reached the appropriate concentration and then they were dispersed in an automated wet dispersion until the obscuration was between 10–20%. Refractive indices of 1.47 and 1.33 were set for oil droplets and deionized water, respectively. The mean particle size was expressed as volume mean diameter (*D_4,3_*).

### 2.6. Rheological Behavior Analysis

#### 2.6.1. Macrorheological Behavior

Rheological behavior was measured using a rheometer (DHR-2, Crawley, UK) equipped with a 40 mm-diameter parallel plate. The apparent viscosities were measured at the shear rate of 0.1–100 s^−1^. We have selected a more suitable 1% strain in the linear viscoelastic region (LVR). The storage modulus G′ and loss modulus G′′ were obtained through frequency sweeps (0.1–10 Hz frequency and 1% strain). In order to evaluate the thermal stability of the HIPEs, the G′ and G′′ were obtained (1 Hz frequency and 1% strain) as the temperature sweep range from 25 °C to 80 °C at a temperature ramp of 5 °C/min, and then maintained at 80 °C for 30 min before being cooled back to 25 °C in the same manner [16].

#### 2.6.2. Micro-Rheological Behavior

A LAB 6 Microrheometer (Formulaction, Toulouse, France) was used to characterize the microrheological properties of HIPEs based on diffusive wave spectroscopy (DWS) technology within 4 h at 25 °C [17]. The mean-square displacement (MSD), elasticity index (EI), and macroscopic viscosity index (MVI) were obtained through Rheosoft Master 1.4.0 software analysis. The motion of the droplet within the sample was quantified as the MSD of the scatterer, which was a direct probe used to obtain the dynamic properties of the droplet embedded in it.

### 2.7. Back-Scattering Light (BS) and TURBISCAN Stability Index (TSI)

HIPEs destabilization phenomena characterized by the BS and TSI were determined through vertical scanning by applying 880 nm pulsed near-infrared light from bottom to top using TURBISCAN LAB Expert (Formulation, Toulouse, France) equipment coupled with TurbiSoft 2.0 software analysis according to the method of Yue et al. [18]. The samples (20 g) were transferred to a measuring cell and scanned for 24 h at room temperature (25 °C).

### 2.8. Application of HIPEs in 3D Printing

The HIPEs were printed using a 3D printer (Shinnove-E Pro, Hangzhou, China). The relevant parameters were as follows. Nozzle height: 0.8 mm; nozzle diameter: 0.84 mm; printing speed: 25 mm/s. A cylindrical shape with a diameter of 20 mm and height of 10 mm was printed.

### 2.9. Processing and Environmental Stress

#### 2.9.1. Centrifugation Stability

Centrifugation at 10,000 rpm for 10 min at 4 °C was applied to the HIPEs to observe the appearance differences and microstructural changes of the samples before and after treatment to evaluate centrifugation stability.

#### 2.9.2. Storage Stability

Storage at 4 °C for 90 days was applied to the HIPEs to observe the appearance differences and microstructural changes of the samples before and after treatment to evaluate storage stability.

#### 2.9.3. Thermal Stability

Heating at 80 °C for 30 min was applied to the HIPEs to observe the appearance differences and microstructural changes of the samples before and after treatment in order to evaluate thermal stability [19]. The rheological behavior of HIPEs during the entire heating and cooling process was also analyzed.

#### 2.9.4. Freeze–Thaw Stability

The HIPEs were frozen in a −20 °C refrigerator for 24 h, thawed at room temperature (25 °C) for 2 h, and cycled 1–3 times according to the actual situation, and then freeze–thaw stability was evaluated by observing the appearance of the samples.

### 2.10. Statistical Analyses

All the measurements were conducted in triplicate, and the data were expressed as mean ± S.D. of all measurements. All diagrams were created with Origin 2021 software.

## 3. Results and Discussion

### 3.1. Appearance and Micromorphology of HIPEs

**Figure 1:**
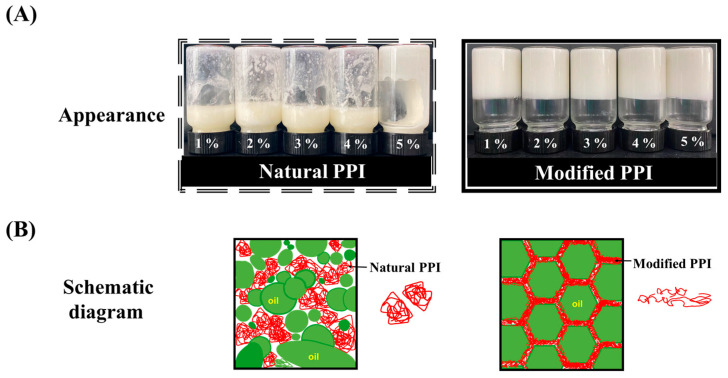
(**A**) Appearance of HIPEs stabilized by natural PPI and modified PPI at different concentrations (1–5 wt%). (**B**) Mechanism schematic diagram of HIPEs stabilized by natural PPI and modified PPI.

As shown in Figure 1A, 1–4 wt% natural PPI-stabilized HIPEs undergo drastic demulsification during shearing. This phenomenon was improved when the concentration increased to 5 wt%, but it showed high flow behavior and phase separation also occurred. However, the HIPEs which were stabilized by MPPI showed a gel-like appearance with good self-supporting properties and no phase separation, indicating that it had the ability to stabilize HIPE. The possible explanation for this phenomenon was that the structure of natural PPI was highly ordered, had a certain rigidity, and presented an agglomeration state, which meant it could not be adsorbed at the interface [4]. As depicted in Figure 1A, the natural PPI aggregates were scattered in the continuous phase and could not play the role of stabilizing the oil droplets. A large number of irregularly shaped oil droplets were free or floating in the system and could not be arranged in order. Under the action of external force, the system would undergo severe coalescence and demulsification. However, the hydrophobic groups and polar sites of the MPPI were exposed and the α-helical content was reduced, thus improving the solubility and emulsification [4]. MPPI with flexible structure could effectively expand and rearrange at the oil–water interface during the shearing process to tightly coat oil droplets and form a compact elastic interface film [4]. Thus, HIPEs with the typical hexagonal oil drop structure of mutual extrusion and orderly arrangement were formed [1,20], as shown in Figure 1A. In order to evaluate the effect of MPPI concentration changes on the interfacial behavior and network structure of HIPEs in more detail, and to reveal the dynamic emulsification process and stabilization mechanism accordingly, the appearance and microstructure were characterized.

**Figure 2:**
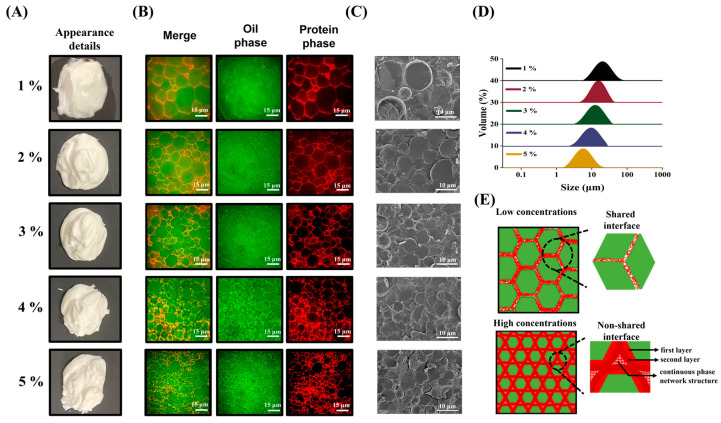
(**A**) Appearance details, (**B**) super-resolution microscopy, (**C**) cryogenic scanning electron microscopy, (**D**) droplet size and (**E**) schematic diagram of emulsification process and stabilization mechanism HIPEs stabilized by 1–5 wt% MPPI.

The appearance was an external manifestation of the altered microstructure and droplet size of the HIPE. In order to observe the surface details more directly, the HIPEs were spread to a flat surface and photographed from above as in Figure 2A. The surface of HIPEs stabilized by 1 wt% MPPI shows a macroscopic oily sheen and relatively poor plasticity. As concentration increased, HIPEs were basically free of oil leakage, and plasticity was enhanced by increase in their surface detail texture, which tends to be stable.

SRM was often used to observe the state of interface distribution and network structure composition of HIPEs [15]. As shown in Figure 2B, the 1 wt% MPPI-stabilized HIPEs showed a typical irregular hexagonal structure. However, the large size of oil droplets (green fluorescence) and the thin interfacial layer shared by adjacent droplets were prone to the occurrence of unstable phenomena such as droplet flocculation and fusion, leading to oil leakage. In the 2–3 wt% stabilized HIPEs, it was observed that some large-sized droplets were surrounded by small-sized droplets, which was associated with the thickening of the local interfacial layer. The droplet size of 4–5 wt% MPPI-stabilized HIPEs was further reduced and uniformly distributed, and a significant enhancement of red fluorescence was observed, representing the formation of bilayer interfacial protein membranes [21,22].

Cryo-SEM demonstrated a similar trend (Figure 2C), which was also reflected in the droplet size results (Figure 2D). In addition to observing dimensional changes, Cryo-SEM could be used to observe the uniform continuity of the interfacial film and the droplet accumulation state. As the concentration increases, the droplet surface gradually becomes smoother and the distance between adjacent droplets seems to increase, which is related to the formation of bilayer elastic interfacial film and network structure [23].

Based on the information above, the dynamic emulsification and stabilization mechanisms of 1–5 wt% MPPI in the formation of HIPEs were further discussed and elaborated, as shown in Figure 2E. The low concentration of MPPI could not completely coat the droplets and form a thin shared interfacial layer, resulting in the formation of unstable HIPEs systems with large droplet size. However, at high concentrations, MPPI was capable of producing homogeneous, continuous, and stable encapsulated droplets that even formed bilayer interfacial films. In addition, unadsorbed MPPI forms a dense 3D network by inter- or intra-molecular interactions entangled in the continuous phase. Consequently, droplets did not need to share the interface film to maintain the stability of the system, and this mechanism was also reflected in the SRM. The double-layer elastic interfacial film and dense network structure inhibit the free motion of droplets to prevent instability [19], and in addition endow HIPEs with good rheological behavior.

### 3.2. Rheological Behavior Analysis

#### 3.2.1. Macrorheological Behavior

**Figure 3:**
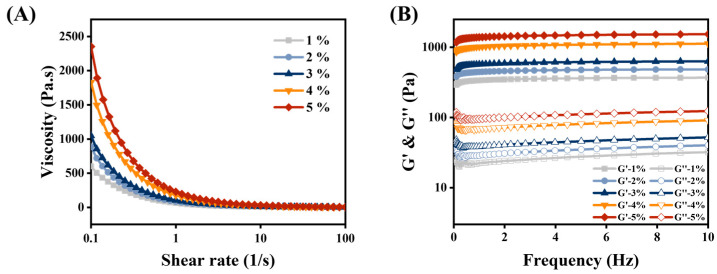
(**A**) Strain sweeps and (**B**) frequency sweep of HIPEs stabilized by 1–5 wt% MPPI.

Macrorheology analyzes the stability and functional properties of HIPEs from an interface perspective. As shown in Figure 3A, all HIPEs exhibited typical shear-thinning behavior, with viscosity decreasing with increasing shear rate [24]. In general, the viscosity of HIPEs was related to droplet size, droplet interactions, and structural changes in the continuous phase [25]. The apparent viscosity of HIPEs was positively correlated with concentration, for the following reasons. Firstly, the increase in MPPI concentration leads to a decrease in droplet size. Secondly, the thicker interfacial layer increases the friction between droplets and inhibits the free-flowing behavior of droplets, resulting in increased viscosity and stability of HIPE. Finally, the unadsorbed MPPI forms a 3D network through molecular cross-linking, which enhances the filling rate of the voids between the droplets, increasing in the viscosity of the continuous phase and the formation of steric hindrance [26].

The frequency sweep reflects the viscoelastic behavior of HIPEs [27]. Figure 3B shows that the storage modulus (G′) behavior dominates in all samples during the frequency sweep, confirming the solid elastic behavior of HIPEs. Furthermore, the storage modulus (G″) was almost 10 times higher than the loss modulus (G″), indicating the formation of a strong 3D network structure [28]. G′ was almost independent of frequency variation, proving that HIPEs stabilized by MPPI were strong gel-like emulsions [13]. G′ increased with increasing MPPI concentration. It is possible that, with the increase in MPPI concentration, the droplet size decreased and became uniformly distributed and closely arranged, which enhanced the network and formed a more highly viscoelastic gel-like soft structure. Then, the interfacial MPPI formed a more stable and dense viscoelastic interfacial film through interaction (hydrophobic interaction) and structural rearrangement. The cross-link bridging between adsorbed and non-adsorbed MPPI and the development of interconnected 3D network structures were also responsible for the high elasticity of the emulsions [29]. In addition, non-adsorbed protein aggregated, filling the gaps of oil droplets were used to support HIPE. These factors contribute to the formation of a gel-like stable network structure with high elasticity.

Macrorheology, too, can be used to evaluate the potential of HIPEs as 3D printing ink [30]. During the printing process, HIPEs utilize shear thinning properties to allow extrusion as well as sufficient viscosity and mechanical strength for supporting layer-by-layer stacking structures in order to produce high-definition printing products [31].

#### 3.2.2. Microrheological Behavior

Microrheology is a rheology that characterizes the microstructure of a sample and obtains the viscoelastic information of the sample by tracing the mean-square displacement trajectory of the Brownian motion scattering particles [22]. Therefore, in this study, microrheology was used to study the interaction relationship between droplet–droplet, droplet–interface protein, and droplet–continuous phase network.

**Figure 4:**
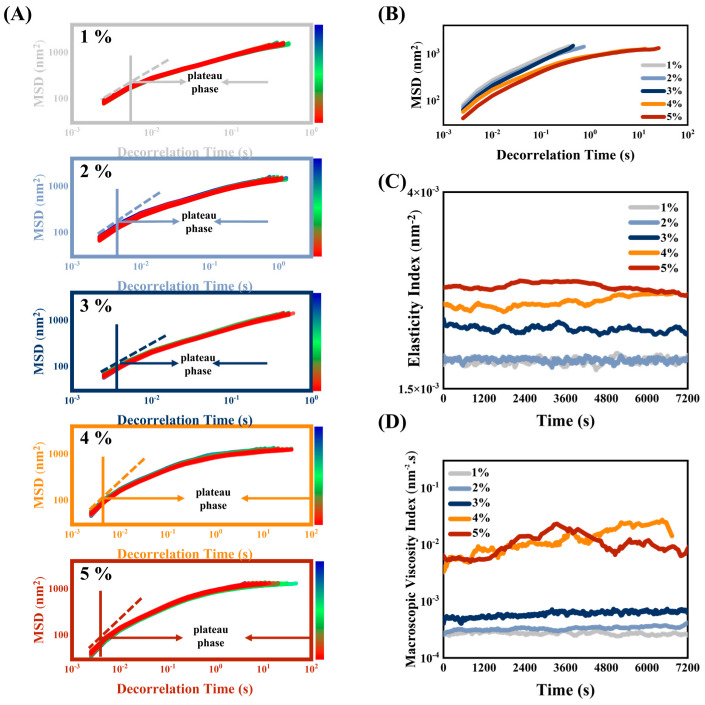
(**A**) Mean-square displacement scanning curve and (**B**) single mean-square displacement single scanning curve at 1 h of HIPEs stabilized by 1–5 wt% MPPI. (**C**) Elasticity index and (**D**) macroscopic viscosity index of HIPEs stabilized by 1–5 wt% MPPI.

If the MSD shows a straight line over time, the sample is a free-moving Newtonian fluid. If the MSD changes with time as a characteristic curve with a plateau region, the sample is a non-Newtonian fluid that cannot move freely (viscoelastic characteristic) [17]. The MSD curves of HIPEs which had been stabilized by MPPI (1–5 wt%) are shown in Figure 4A, all showing viscoelastic characteristics. The initial MSD linear region was related to the viscosity of the solvent. [22]. Then, the droplets were trapped in a “cage mesh” composed of the viscoelastic material microstructure, and the slope of the MSD curve began to decrease into a plateau phase. This stage was used to discuss the elastic behavior of HIPEs by characterizing the structural features of the interfacial film that wraps the droplets [32,33]. The elasticity ondex (EI) corresponds to the inverse of the height of the MSD platform, which was the inverse of the distance required for a droplet to touch the “cage mesh” [18]. A lower MSD plateau height represents a smaller size of the “cage mesh” or thicker and denser HIPEs, which means less space for the droplet to move freely, indicating higher elasticity of HIPE. As shown in Figure 4B,C, with the increase in MPPI concentration, the MSD platform height decreased and EI increased, indicating that a double-layer elastic interface protein film was gradually formed, and a sufficient amount of unadsorbed proteins in the continuous phase were intertwined to form a dense 3D network that prevents the droplets from merging or deforming, thus endowing HIPEs with high elasticity. With time, the droplets escape from the “cage net” and the MSD curve rises linearly again. Interestingly, the plateau phase of HIPEs stabilized at 4 wt% and 5 wt% MPPI seems to be sustained, indicating that the movement of droplets in the system was very slow, which in turn implies the formation of highly stable HIPEs. The MVI, which is the inverse of the slope of the MSD curve after the plateau phase, quantifies the macroscopic viscosity at zero shear and the velocity of the droplet [34]. The lower the slope is, the slower the droplet motion will be, and these phenomena correspond, physically, to the higher macroscopic viscosity of HIPEs. The same trend was observed for MVI and EI in Figure 4D. As MPPI concentration increased, droplet size decreased and droplet-to-droplet packing was more tightly packed, resulting in the formation of highly stable viscoelastic HIPEs that were resistant of the deformation network system, which facilitates referencing in 3D printing. The EI and MVI of HIPEs were consistent with the results obtained for G′ (Figure 3B) and the apparent viscosity (Figure 3A) in macrorheology, respectively. In addition, the curves of EI and MVI values for both 4 wt% and 5 wt% MPPI intersected throughout the scan, which indicates that their states are similar.

### 3.3. Physical Stability Evaluation

Within 24 h, the sample was repeatedly scanned from bottom to top, and data of backscattered light and transmitted light (i.e., BS and TSI) were collected to obtain a map characterizing the stability of the sample [32]. According to the highly concentrated characteristics of the prepared HIPEs, BS was selected to analyze and characterize the homogeneity, droplet size, and concentration of the system, thereby judging the stability of the system [35].

**Figure 5:**
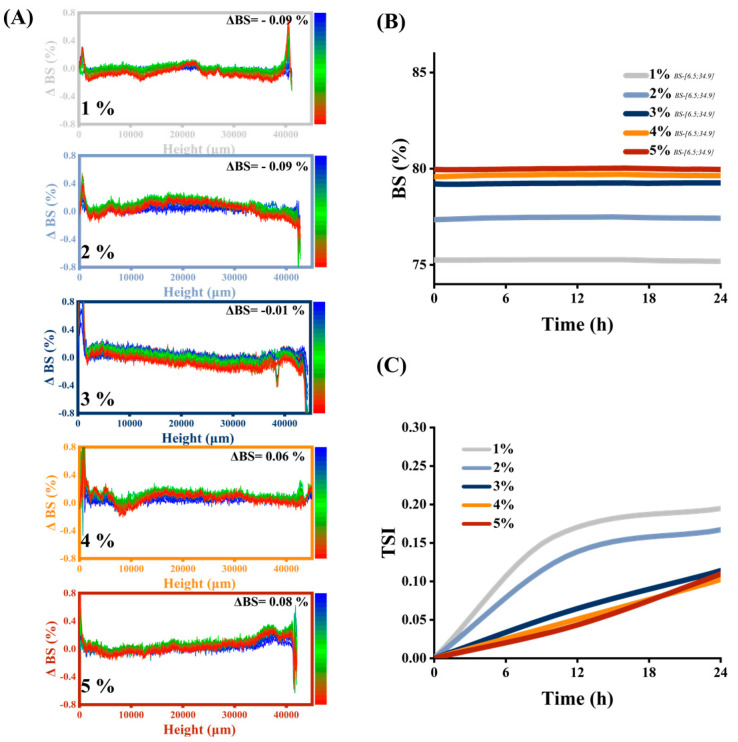
(**A**) Backscattered light rate (%) of change spectrum, (**B**) backscattered light (%) at the same coordinates and (**C**) TURBISCAN stability index of HIPEs stabilized by 1–5 wt% MPPI.

The ΔBS scan patterns of HIPEs stabilized by MPPI (1–5 wt%) are shown in Figure 5A. The horizontal axis represents the sample height (0 to 40 mm), the left vertical axis represents the rate of BS change, and the right vertical axis represents the scan time. The BS value was related to the droplet size. In HIPE systems with high turbidity, the intensity of the backscattered light follows the Mie scattering theory and decreases with the increase in droplet size [36]. During the entire process, the BS fluctuation of all samples was less than 0.2%, indicating that the internal microstructure and distribution state of HIPEs stabilized by MPPI did not change drastically and had high stability. The HIPEs stabilized with 1–3 wt% MPPI had ΔBS < 0, indicating that a certain degree of contact deformation or even fusion occurred between the droplets of HIPEs during the test time, resulting in a decrease in the BS value [18]. The BS in the 1–2 wt% groups decreased by 0.09%, indicating that the MPPI concentration was not sufficient to completely coat the droplets. In addition, it shows that the oil–water interface was always undergoing dynamic change and that the adsorption and desorption of proteins continues to occur [32]. The BS in the 3 wt% group decreased by 0.01%, proving that the MPPI adsorbed in the second layer played a protective role on the MPPI closer to the oil–water interface, that the exchange between adsorbed and non-adsorbed MPPI was reduced, and that HIPEs developed towards a more stable direction [36]. The ΔBS > 0 of the 4–5 wt% group indicated that the droplet size of HIPEs remained stable or even decreased during the test time. As MPPI concentration increased, the bilayer elastic interfacial film and the continuous phase network structure were gradually formed, the interaction between MPPI was strengthened, the droplets were stably encapsulated and fixed in the network, and the size and morphology would not change substantially. Furthermore, a certain period of storage to allow interactions between MPPI molecules to accomplish tighter adsorption and denser 3D network structure formation resulted in a droplet size reduction. In Figure 5B, the BS values were obtained by scanning at the same coordinates. The BS value was proportional to the protein concentration, indicating that the droplet size decreased and the distribution was uniform as the protein concentration increased.

TSI was used to characterize the overall stability of HIPEs, with high TSI values representing system instability [37]. The lowest TSI values occurred at HIPEs stabilized by 5 wt% MPPI, demonstrating that thy had the highest stability. Furthermore, the higher viscosity and elasticity of HIPEs inhibited droplet migration and improved stability, which was consistent with the results of rheological analysis.

### 3.4. Application of HIPEs in 3D Printing

Based on the above results on microstructure and rheological properties, the potential of HIPEs in 3D printing was further discussed. All the HIPEs were smoothly extruded from the nozzle during printing owing to their shear thinning properties [31].

**Figure 6:**
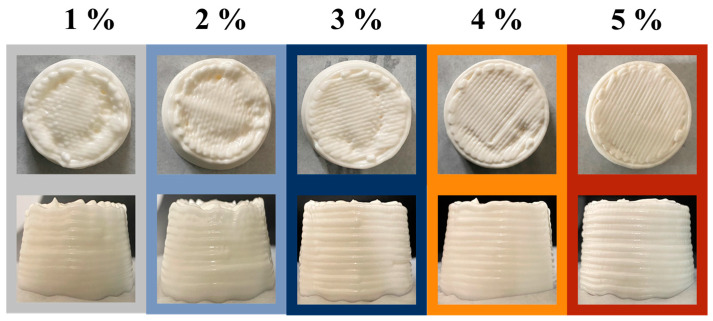
The visual appearance of 3D printed samples of HIPEs stabilized by 1–5 wt% MPPI.

As illustrated in Figure 6, cylinders printed with HIPEs stabilized by 1–2 wt% MPPI showed poor shape fidelity, with an obvious collapse at the top, trapezoidal sides, sagging structures, obvious layer fusion, and even faults. As the protein concentration increased, the degree of collapse decreased, and the stacked layers were more easily distinguished, but there were still some minor defects. The 5 wt% group shape had high definition and high resolution, the collapse largely disappeared, the surface was smooth, and the structure a certain degree of self-supporting ability. The actual printed shape was close to the modeled shape, and the details were perfect. This change was associated with an improvement in its rheological behavior, as rheology was a bridge between edible inks and their 3D printability [35].

### 3.5. Processing and Environmental Stress

#### 3.5.1. Centrifugation Stability

**Figure 7:**
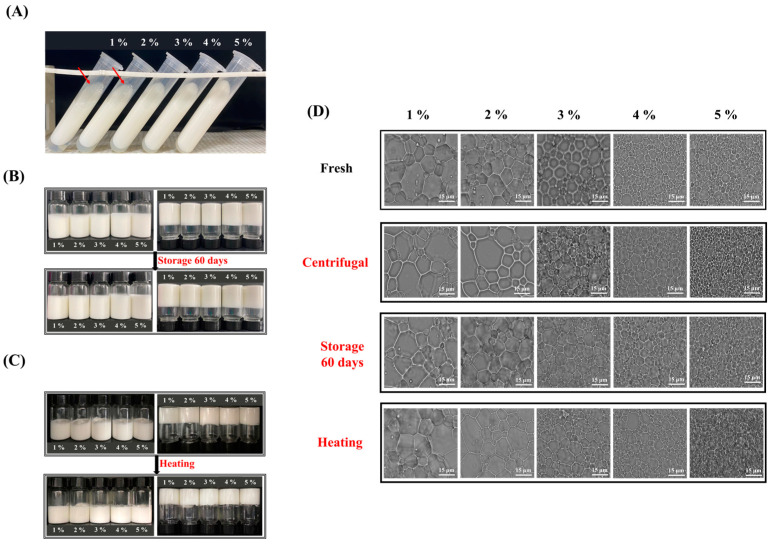
Appearance of HIPEs stabilized by 1–5 wt% MPPI after (**A**) centrifugation, (**B**) storage, and (**C**) heat treatment. (**D**) Optical microscope of HIPEs stabilized by 1–5 wt% MPPI before and after centrifugation, storage, and heat treatment.

The stability of HIPEs was further evaluated by centrifugation, storage, heating, and freeze–thaw treatments. HIPEs with good centrifugation stability were beneficial to maintaining structure and properties in practical processing. The appearance of HIPEs stabilized by MPPI after centrifugation is shown in Figure 7A. Some degree of oil leakage occurred in the 1–2 wt% group but was not observed in the 3–5 wt% group. The interfacial film formed by the low concentration of MPPI was thin and could not resist the forced collision under the centrifugal action, resulting in the rupture of the interfacial film and oil being removed (Figure 7D). With the increase in MPPI concentration, the thickness of the interfacial film was superimposed onto the double layer, and the intermolecular interaction was strengthened to form a network structure with a mechanical barrier effect, which doubles the protection of the droplets to avoid oil breakage. In addition, the high centrifugation stability enables the manufacture of ultra-high internal phase emulsions by centrifugation. Zhang et al. [26] used the centrifugation method to produce an ultra-high internal phase emulsion with good stability under gliadin/sodium carboxymethyl cellulose complex particles (GCCPs) of a relatively low concentration (0.3–1.5 wt%) of an emulsifier by adjusting the centrifugal speed (2000–12,000 r/min). Its internal phase could reach a maximum of 90.28%. Furthermore, it was observed that the water phase at the bottom decreased with the increase in MPPI concentration after centrifugation, indicating that the HIPEs system had a stronger ability to bind water, which was related to the interaction between molecules and the formation of a dense network structure. In addition, the increased viscosity of HIPEs may have limited the precipitation of the water layer during centrifugation [30]. High concentrations of MPPI enhanced the centrifugation stability of HIPEs. However, it was found that the bottom aqueous layer after centrifugation became gradually turbid with the increasing MPPI concentration. The adsorbed MPPI would undergo a certain degree of unfolding at the interface, resulting in the exposure of hydrophobic groups and the interaction between molecules through hydrogen bonding to form a more elastic interface film. The result of this is that MPPI could be adsorbed tightly and stably and would not be easily removed from the system [4]. Therefore, the centrifuged material may comprise more of the unadsorbed protein thrown out of the emulsion system. First, it was demonstrated that some unadsorbed proteins were indeed used to form a network structure in the continuous phase in order to provide steric hindrance and confine oil droplets, thereby enhancing the stability of HIPEs. Second, it was not necessary to increase the protein concentration on the basis of 5 wt% in order to further improve the performance and stability of HIPEs because the contribution rate was very low.

#### 3.5.2. Storage Stability

No samples showed any significant changes in appearance and microstructure compared with fresh samples after 90 days of storage, as shown in Figure 7B,D, demonstrating the high storage stability of HIPEs stabilized by MPPI, which has the ability to improve shelf life in food applications [16]. Although the low concentration of MPPI-stabilized HIPEs had a single-layer interface film shared by adjacent droplets, limited ability to coat droplets, and poor centrifugal stability, it could still remain stable for a long time without the presence of a strong external force. In addition to being able to maintain structural and property stability, Zhang et al. [38] demonstrated that HIPEs stabilized by quinoa protein isolate exhibit increased storage modulus and interfacial layer thickness after storage, possibly because of enhanced protein–oil droplet and protein–protein interactions.

#### 3.5.3. Thermal Stability

**Figure 8:**
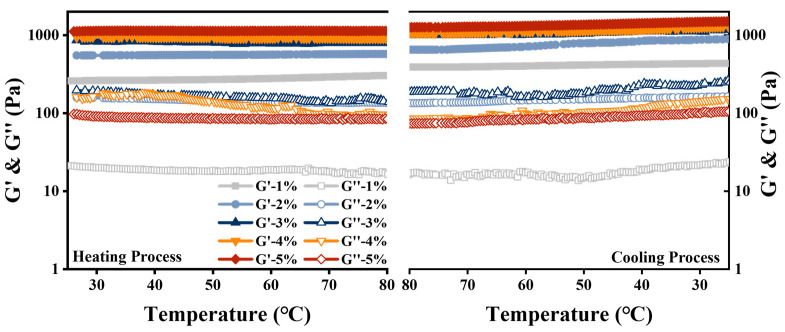
The response of storage modulus and loss modulus to temperature change of HIPEs stabilized by 1–5 wt% MPPI.

Thermal stability may affect the application of HIPEs in food processing, we consequently investigated the effect of heating on the appearance, microstructure, and rheological properties of HIPEs. The appearance of the HIPEs remained stable after heating, with some tiny pores, which were caused by the gas escaping because of heating (Figure 7C). Droplet size and microstructure did not change significantly in Figure 7D. The response of G′ and G″ to temperature change at 1% strain and frequency of 1 Hz are shown in Figure 8. The G’ of HIPEs stabilized by various MPPI concentrations remained stable during heating, indicating its excellent thermal stability. The G′ of all HIPEs increased significantly during cooling, owing both to the formation of aggregates after denaturation and the unfolding of MPPI at high temperature, which supported the network structure and improved the mechanical strength [39]. Similar phenomena had also been observed in studies of other material-stabilized HIPEs such as gliadin [26] and gelatin [19].

#### 3.5.4. Freeze–Thaw Stability

**Figure 9:**
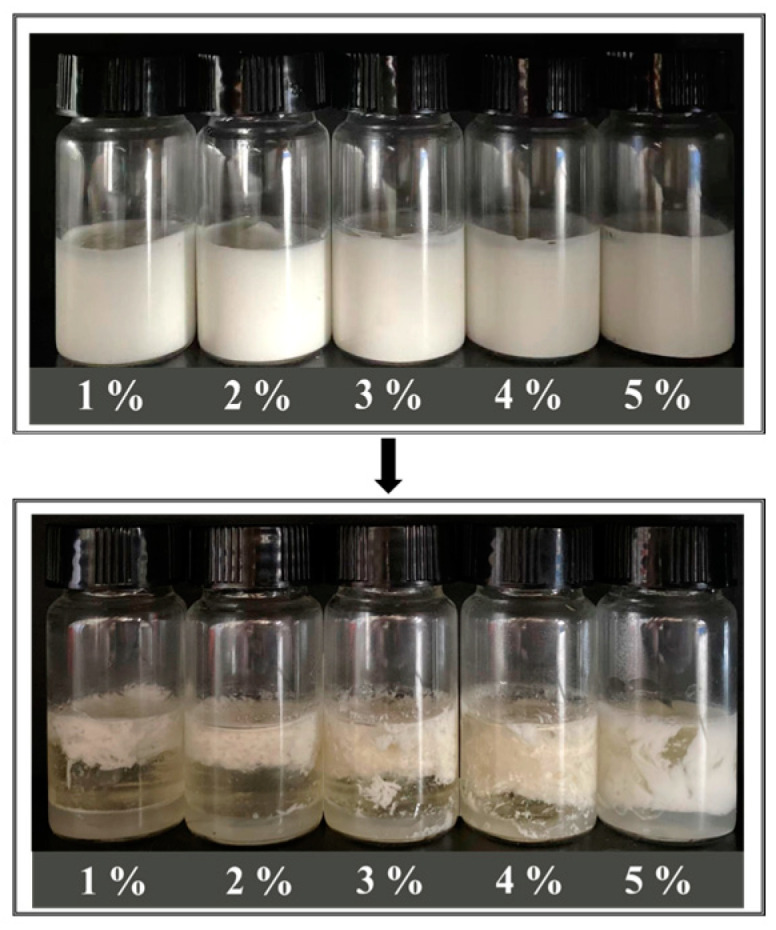
Appearance of HIPE stabilized by 1–5 wt% MPPI before and after one freeze–thaw cycle.

As shown in Figure 9, HIPEs stabilized by MPPI were completely demulsified after one freeze–thaw cycle, demonstrating their poor freeze–thaw stability. First, a large number of ice crystals formed during the freezing pierced the interfacial film, resulting in severe oil leaching after thawing [16]. Furthermore, the droplets were forced to aggregate in the unfrozen aqueous phase, causing the droplets to coalesce [19]. According to the experiments in the work of Xu et al. [40], HIPEs stabilized by rapeseed protein isolate were also strongly demulsified after a single freeze–thaw cycle. Although demulsification occurred in all HIPEs, the degree of phase separation appeared to decrease as the MPPI concentration increased. The thicker interfacial film and dense network formed at higher MPPI concentrations slightly inhibited droplet coalescence and piercing behavior.

In conclusion, HIPEs stabilized by MPPI of high concentrations had higher viscoelasticity and plasticity, better stability (centrifugation, storage, and heating), and a wider range of applications.

## 4. Conclusions

This study elucidates the emulsification process and stabilization mechanism of MPPI-stabilized HIPEs by discussing the interfacial distribution state and network structure composition. It additionally demonstrates that the viscoelasticity and stability (centrifugation, storage, and heat stability) of HIPEs improve with the increase in MPPI concentration. This effect was attributed to the formation of a bilayer elastic interfacial membrane and three-dimensional network structure, as well as to enhanced protein intra- and intermolecular interactions. The HIPEs constructed in this study may be useful in developing highly viscous and malleable products (e.g., 3D printing or fat simulants) for applications in the food industry. However, in view of the poor freeze–thaw stability and the limitations imposed by the protein’s own structure, further studies are needed to circumvent the disadvantages and amplify the advantages of this method in order to expand the application of HIPEs in a wider range of industries.

## Data Availability

The data presented in this study are available in [high internal phase emulsions stabilized by ultrasound com-bined with pH-shifting modified pea protein isolate: Micro-morphology, rheology, and physical stability].
